# Optineurin-mediated mitophagy as a potential therapeutic target for intervertebral disc degeneration

**DOI:** 10.3389/fphar.2022.893307

**Published:** 2022-08-29

**Authors:** Zhilei Hu, Yu Wang, Xiaoxin Gao, Yuyao Zhang, Chenhao Liu, Yu Zhai, Xian Chang, Haiyin Li, Yueyang Li, Jinhui Lou, Changqing Li

**Affiliations:** ^1^ Department of Orthopedics, Xinqiao Hospital, Army Military Medical University, Chongqing, China; ^2^ Department of Ophthalmology, Southwest Hospital, Army Military Medical University, Chongqing, China

**Keywords:** OPTN, mitophagy, intervertebral disc degeneration, nucleus pulposus cell, senescence

## Abstract

Low back pain is thought to be mainly caused by intervertebral disc degeneration (IVDD), and there is a lack of effective treatments. Cellular senescence and matrix degradation are important factors that cause disc degeneration. Mitochondrial dysfunction induced by oxidative stress is an important mechanism of cellular senescence and matrix degradation in the nucleus pulposus (NP), and mitophagy can effectively remove damaged mitochondria, restore mitochondrial homeostasis, and mitigate the damage caused by oxidative stress. Optineurin (OPTN) is a selective mitophagy receptor, and its role in intervertebral disc degeneration remains unclear. Here, we aimed to explore the effect of OPTN on H_2_O_2_-induced nucleus pulposus cell (NPCs) senescence and matrix degradation in a rat model of disc degeneration. Western blot analysis showed that OPTN expression was reduced in degenerative human and rat nucleus pulposus tissues and increased in H_2_O_2_-induced senescent NPCs. OPTN overexpression significantly inhibited H_2_O_2_-induced senescence and increased matrix-associated protein expression in NPCs, but OPTN knockdown showed the opposite effect. As previous reports have suggested that mitophagy significantly reduces mitochondrial damage and reactive oxygen species (ROS) caused by oxidative stress, and we used the mitophagy agonist CCCP, the mitophagy inhibitor cyclosporin A (CsA), and the mitochondrial ROS (mtROS) scavenger mitoTEMPO and confirmed that OPTN attenuated NPCs senescence and matrix degeneration caused by oxidative stress by promoting mitophagy to scavenge damaged mitochondria and excess reactive oxygen species, thereby slowing the progression of IVDD. In conclusion, our research suggests that OPTN is involved in IVDD and exerts beneficial effects against IVDD.

## Introduction

Low back pain (LBP), which is mainly caused by intervertebral disc degeneration (IVDD), has become a global health issue that severely affects the quality of life of patients, leads to disability and imposes serious socioeconomic burdens (Vos et al., 2017). Nucleus pulposus cells (NPCs) senescence and extracellular matrix degradation are important molecular mechanisms of IVDD (Feng et al., 2016; Feng et al., 2017). As intervertebral discs age and degenerate, senescent NPCs accumulate, and matrix anabolism-related protein levels decrease, causing the NP to gradually lose its ability to maintain matrix homeostasis and self-renew. Oxidative stress is a common degeneration-related pathological factor linked to a wide range of diseases, such as glaucoma (Fan Gaskin et al., 2021), osteoarthritis (Tudorachi et al., 2021), and neurodegenerative diseases (Forman and Zhang, 2021). In normal intervertebral discs, reactive oxygen species (ROS) participate in physiological processes by acting as signaling molecules; in degenerated human discs, abnormally high ROS levels lead to disc lesions (Feng et al., 2017). Therefore, inhibiting oxidative stress and restoring redox homeostasis in NPCs may be effective measures to slow IVDD.

Mitochondria are not only the most important sources of energy in the cell but also major sources of intracellular ROS (Zorov et al., 2014). Mitochondrial dysfunction is the main cause of excessive ROS production, and excess ROS attack and damage mitochondria, causing further production of mitochondrial ROS (mtROS), which leads to cellular dysfunction. As a selective form of autophagy, mitophagy maintains the dynamic balance of mitochondria by eliminating damaged mitochondria (Gustafsson and Dorn, 2019). Mitophagy insufficiency results in untimely clearance of damaged mitochondria from the cell, thereby increasing mtROS production. Abnormal mitophagy is implicated in a wide range of diseases, including Parkinson’s disease (Xia et al., 2020), heart disease (Shao et al., 2020) and osteoporosis (Guo et al., 2021). Mitophagy was recently shown to play an important role in slowing the progression of IVDD (Wang Y et al., 2018; Zhang et al., 2018; Xie et al., 2019; Xu et al., 2019). Considering that oxidative stress and mitophagy are implicated in the pathogenesis of IVDD, enhancing mitophagy may exert a beneficial effect against and delay the progression of IVDD.

Optineurin (OPTN) is a highly conserved protein that plays a role in vesicular transport (Schwamborn et al., 2000) and NF-KB signaling (Nakazawa et al., 2016; Montecalvo et al., 2017), and the discovery of mutations in the OPTN gene that are associated with several familial diseases, such as OPTN^E478G^, which is related to amyotrophic lateral sclerosis (ALS) (Wong and Holzbaur, 2014) and OPTN^E50K^, which is associated with glaucoma, has caused researchers to pay more attention to OPTN (Rezaie et al., 2002; Aung et al., 2005). Importantly, OPTN was identified as a mitophagy receptor and found to contain a ubiquitin-binding domain that is capable of binding polyubiquitinated substrates and translocating them to the autophagosome and eventually to the lysosome via the LC3-interacting domain (Qiu et al., 2021). However, the role of OPTN in IVDD is poorly understood.

In the present study, H_2_O_2_-induced oxidative stress was used to model the pathological process of mitochondrial damage and NPCs senescence to investigate the regulatory role of OPTN-mediated mitophagy in NPCs senescence and extracellular matrix homeostasis. Finally, the therapeutic effects of OPTN were evaluated using a needle puncture-induced IVDD rat model. Our study highlights the mechanism by which OPTN mitigates disc degeneration and explores the potential of OPTN as a therapeutic target for IVDD.

## Materials and methods

### Ethics statement

The collection of human NP tissues and related experiments were approved by the Ethics Committee of the Second Hospital of the Army Medical University.

All surgical, therapeutic and postoperative animal care procedures were carried out in strict compliance with the Laboratory Animal Welfare and Ethics Committee of the Third Military Medical University (AMUWEC20193008).

### Reagents and antibodies

MitoTEMPO, H_2_O_2_, CCCP, and collagenase II were obtained from Sigma (St. Louis, MO, United States). Primary antibodies against P16 and LC3B were obtained from Abcam (Cambridge, United Kingdom). Primary antibodies against OPTN, Parkin, and PINK1 were purchased from Santa Cruz Biotechnology (Dallas, TX, United States). Primary antibodies against β-actin, P21, TIMP1, Aggrecan, Collagen II, MMP-1, and P62 were provided by Proteintech (Wuhan, Hubei, China). Cyclosporin A (CsA) was obtained from Selleck (Houston, TX), and 4′,6-diamidino-2-phenylindole (DAPI) was acquired from Beyotime (Shanghai, China).

### Collection of human NP specimens

To study the connection between OPTN and the severity of IVDD, NP tissue was collected from 20 patients with IVDD (3 males and 2 females, aged 18–40 years, grade II; 4 males and 1 female, aged 16–39 years, grade III; 3 males and 2 females, aged 17–40 years, grade IV; 3 males and 2 females, aged 20–40 years, grade V) according to Pfirrmann grading criteria and used for further experiments (Pfirrmann et al., 2001). In our study, the patients from which NP tissue was collected during surgery did not have other diseases associated with IVDD. The collected NP tissues were lysed to extract proteins for Western blotting.

### Collection of rat NP tissue and NPCs isolation and culture

Gelatinous NP tissue was collected from the tails of 1-, 2-, and 20-month-old rats (Sprague–Dawley rats, male) under aseptic conditions. NP tissue was collected from 1- and 20-month-old rats, and protein was extracted for Western blotting. As described in a previous study (Chen et al., 2020; Lin et al., 2020), NP tissues from two-month-old rats were minced under a dissecting microscope, and the minced NP tissues were incubated in 0.2% collagenase II (St. Louis, MO, United States) for 2 h at 37°C. After being washed twice, the tissues were transferred to T25 flasks containing DMEM/F12 (BI, Kibbutz Beit-Haemek, Israel) supplemented with 10% fetal bovine serum (BI, Kibbutz Beit-Haemek, Israel) and antibiotics (1% streptomycin/penicillin) and incubated at 5% CO_2_ and 37°C. The medium was changed every 3 days. The cells were digested with 0.25% trypsin-EDTA (Gibco, Invitrogen) when they reached 80% confluence and were then passaged. Third-generation NPCs were used for subsequent experiments.

### Cell treatment protocol

To study OPTN expression levels in NPCs during oxidative stress, H_2_O_2_ was used to induce cellular oxidative damage (Lin et al., 2021a; Cheng et al., 2021). NPCs were exposed to different concentrations of H_2_O_2_ (0, 200, 400, 500, 600, 800 μM) for 2 h. The optimal conditions for inducing cellular senescence were determined by treating NPCs with the same concentration of H_2_O_2_ (400 μM) for different times (0, 0.5,1, 2, 4, 6 h). To assess mitophagy, NPCs were treated with or without H_2_O_2_ and then with or without CCCP (30 μM, 1 h). Then the mitophagy inhibitor CsA (10 μM, 1 h) and MitoTEMPO (200 μM, 2 h) were added, and the cells were incubated before subsequent assays.

### Mitophagy assay

Lentiviral constructs expressing tandem mCherry-eGFP-FIS1 (GeneChem, China) were used to transfect the NPCs. Briefly, 1 × 10^5^ NPCs were seeded on glass coverslips in 12-well plates and cultured at 37°C until the cells reached 30–50% confluence. After polybrene was added to the medium, lentiviruses expressing mCherry-eGFP-FIS1 were added at a multiplicity of infection (MOI) of 10, 25, or 50 according to the manufacturer’s instructions. After 48 h, the NPCs transfected with the mCherry-eGFP-FIS1 lentiviruses were used for follow-up experiments. Red particles, which represented mitolysosomes, were counted, and the data were statistically analyzed.

### Lentiviral transfection

An overexpression lentivirus and small hairpin RNAs targeting OPTN were purchased from GeneChem (Shanghai, China). The OPTN-RNAi target sequence was GAG​CTG​CTG​CAG​CAA​ATG​AAA, and the control sequence was TTC​TCC​GAA​CGT​GTC​ACG​T. When the cells reached 30–50% confluence, they were transfected with the lentivirus. After 12–16 h, the medium was replaced. Forty-8 hours later, the cells were screened with medium containing puromycin. When the cells reached 80% confluence, the transfected NPCs were passaged and cultured with medium containing puromycin for subsequent experiments.

### Western blotting

Total protein was extracted from NP tissues and cells using precooled radioimmunoprecipitation analysis (RIPA) lysis buffer containing 1 mM phenylmethane sulfonyl fluoride (PMSF). The protein concentrations were measured with a BCA protein assay kit (Beyotime, Shanghai, China). Then, the proteins were separated by sodium dodecyl sulfate–polyacrylamide gel electrophoresis (SDS–PAGE) and transferred to polyvinylidene difluoride (PVDF) membranes (Millicon, United States). The PVDF membranes were blocked with 5% nonfat dry milk for 2 h and then incubated with primary antibodies against OPTN (1:200), P16 (1:2000), LC3B (1:1000), Parkin (1:500), β-actin (1:5000), P21 (1:1000), TIMP1 (1:500), Aggrecan (1:500), Collagen II (1:500), MMP-1 (1:500), and P62 (1:1000) overnight at 4°C. The blots were then washed with Tris-buffered saline/Tween 20 (TBST) three times for 10 min each and incubated with corresponding secondary antibodies for 2 h. After the blots were washed with TBST, the bands were visualized and quantified using an electrochemiluminescence kit (1705060, Bio-Rad, CA, United States), a gel imaging system (Bio-Rad, CA, United States) and Image Lab 3.0 software (Bio-Rad, United States).

### MitoSOX assay

Intracellular mtROS levels were measured using MitoSOX (Ali et al., 2021). Briefly, rat NPCs were seeded in a six-well plate and treated as described previously. Then, the NPCs were incubated with 5 μM MitoSOX for 20 min at 37°C, washed twice with PBS, and incubated with Hoechst 33342 solution for 15 min at 37°C. Staining was quantified using a fluorescence microscope (Olympus IX73, Tokyo, Japan).

### Mitochondrial membrane potential (MMP) analysis

The MMP was measured using MitoTracker Red CMXRos (C1049B Beyotime, Biotechnology, Shanghai, China), which accumulates in live cells in an MMP-dependent manner to stain mitochondria. NPCs were incubated with 200 nM MitoTracker Red for 30 min at 37°C and then stained with Hoechst 33342 dye at 37°C for 15 min. A fluorescence microscope (Olympus IX73, Tokyo, Japan) was used to observe the staining results, and ImageJ/Fiji (NIH, Bethesda, MD, United States) was used to quantify the results.

### Sa-β-gal staining

Sa-β-gal staining was performed using the Cellular Senescence β-Galactosidase Staining Kit (C0602 Beyotime Biotechnology, Shanghai, China) according to the manufacturer’s instructions. Briefly, NPCs were treated in six-well plates, washed once with PBS, and fixed with β-galactosidase staining solution for 15 min at room temperature. The cells were washed 3 times with PBS for 3 min each. One milliliter of staining working solution was added to each well, and the cells were incubated overnight at 37°C. Images were taken using an Olympus IX71 microscope, and SA-β-gal-positive cells were quantified for statistical analysis.

### Rat IVDD model

Twenty-four 8-week-old male Sprague–Dawley rats were randomly divided into 3 groups: the sham group (skin excision but no puncture of the intervertebral space), Lv-ctrl group (puncture of the intervertebral space + injection of empty virus), and Lv-OPTN group (puncture + OPTN overexpression virus injection). A rat model of IVDD was established as described previously (Zhang et al., 2009). After the rats were anesthetized with 2% (w/v) sodium pentobarbital (40 mg/kg), the tail disc (CO4/5) was localized by palpation and labeled. After being sterilized, a puncture needle (27 G) was passed vertically through the back skin into the annulus fibrosus (AF) at a depth of 4 mm, rotated 360° and left in the disc for 1 min. The health of the rats was monitored daily after surgery, and the rats were sacrificed at 8 weeks for follow-up experiments.

### Intervertebral space height measurement

Rats that had undergone *in vivo* experiments were X-rayed at 0 and 8 weeks after surgery using a multimode small animal live imaging system (Xtremell, Bruker, Belgium). Briefly, after being anesthetized by intraperitoneal injection of 2% pentobarbital, the rats were fixed in the prone position on an imaging table, and the disc heigh index (DHI) was measured as previously described (Issy et al., 2013).

### Magnetic resonance imaging (MRI)

MRI was performed with a 7.0 T MRI device (Bruker PharmaScan, Germany) at 0 and 8 weeks postoperatively. Structural and signal differences in the tail discs were assessed with sagittal T2-weighted images. Magnetic resonance parameters were selected according to previous reports (Pfirrmann et al., 2001), and a researcher blinded to the groups evaluated the MRI images in accordance with the Pfirrmann grading criteria.

### Histopathological staining

After MRI and X-ray, the rats were sacrificed by intraperitoneal injection of an overdose of sodium pentobarbital, and the intervertebral discs and adjacent caudal vertebrae of the segments of interest were collected. The specimens were fixed in paraformaldehyde, decalcified, dehydrated embedded in paraffin and cut into sections at a thickness of 5 μm for hematoxylin-eosin and safranin O-fast green staining, and disc degeneration was evaluated. The histological scores were determined in a blinded manner according to histological grading criteria (Tam et al., 2018) (0-5: normal; 6-11: moderate; 12-15: for severe).

### Statistical analysis

Statistical analysis of the raw data was performed using the statistical software SPSS 23.0 (IBM, United States) and GraphPad Prism 8.00 (GraphPad Software, United States), and the results are expressed as the mean ± S.D. Two sets of data were statistically analyzed using Student’s t test. Multiple sets of data were analyzed by one-way analysis of variance (ANOVA) followed by Tukey’s test. Nonparametric data were analyzed by the Kruskal–Wallis H test. A *p* value <0.05 was considered statistically significant.

## Results

### OPTN expression is decreased in human and rat degenerated NP tissues and increased in H_2_O_2_-treated NPCs

**FIGURE 1:**
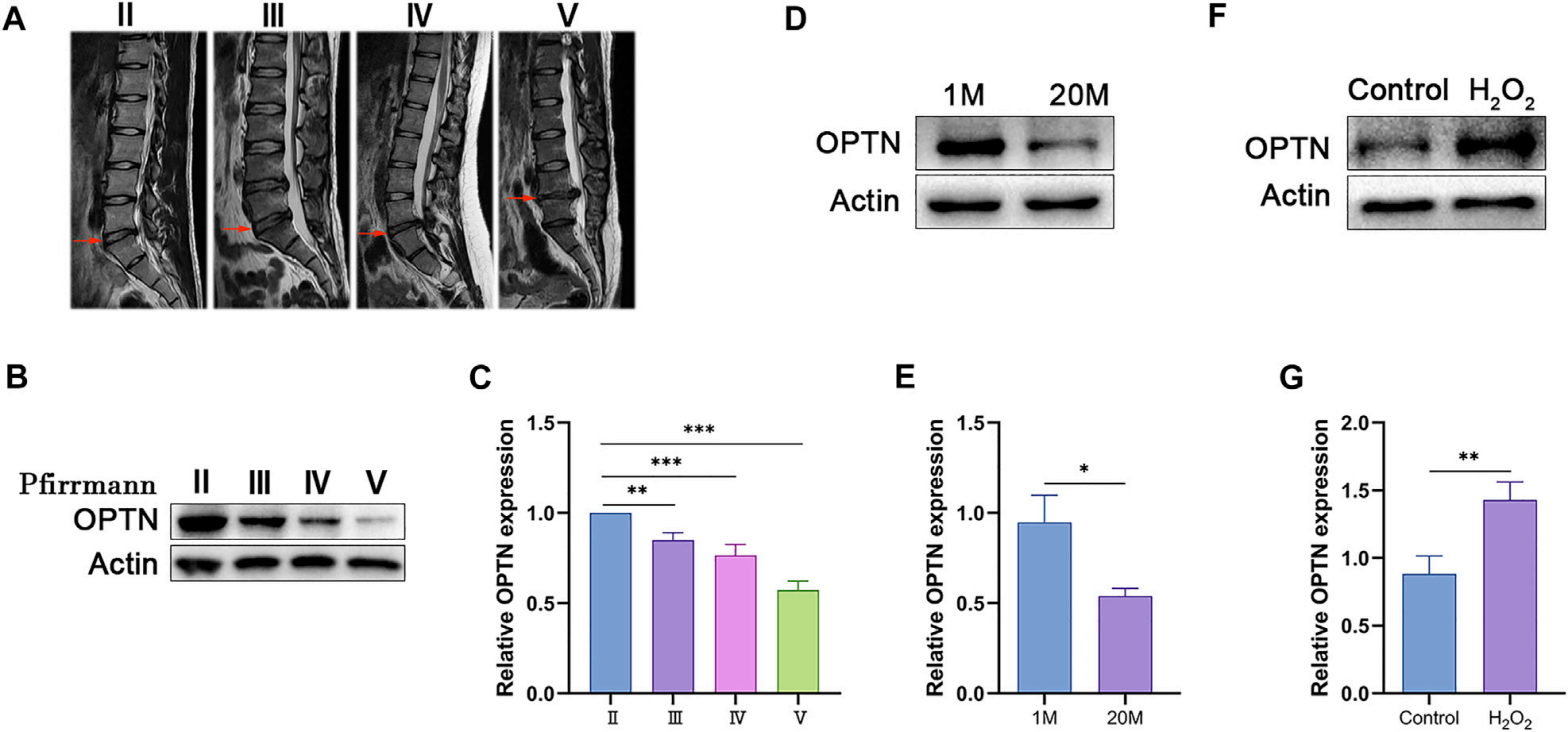
OPTN level decreased in degenerated NP tissue and increased in H_2_O_2_-teated rat NPCs. **(A)** different degrees of IVDD MRI images of patients. **(B–C)** Western blot and quantification of OPTN in human NP tissue. **(D–G)** Western blot and quantification of OPTN in rat NP tissues and H_2_O_2_-teated NPCs. Experiments involving human NP specimen were performed as means ± SD of 5 times in duplicates. Experiments involving rat NP specimen were performed as means ± SD of 3 times in duplicates. *p < 0.05, **p < 0.01, ***p < 0.001, ns *p* > 0.05.

To investigate whether OPTN expression is related to IVDD, we collected NP tissues from five patients with Pfirrmann grade II-V IVDD and measured OPTN expression levels. We also collected caudal NP tissues from young and old rats. As shown in Figures 1A–E, the expression level of OPTN decreased as disc degeneration increased in both human and rat NP specimens. We examined OPTN expression in H_2_O_2_-treated rat NPCs; H_2_O_2_ was used to mimic oxidative stress-induced NPCs senescence, and the cells were treated with 400 μM H_2_O_2_ for 2 h, which was selected as the optimal conditions (Supplementary Figures S1A, B). H_2_O_2_-induced oxidative stress increased OPTN expression in NPCs (Figures 1F,G). As shown in Supplementary Figure S1D, we found that oxidative stress decreased proteoglycan and Collagen II levels, increased MMP-1 expression and decreased TIMP1 expression in NPCs. These results suggest that H_2_O_2_-induced oxidative stress exacerbates cellular senescence and matrix degeneration in rat NPCs. In summary, our results indicate that OPTN may be involved in the pathological process of IVDD.

### OPTN regulates senescence and matrix degradation in H_2_O_2_-treated NPCs

**FIGURE 2:**
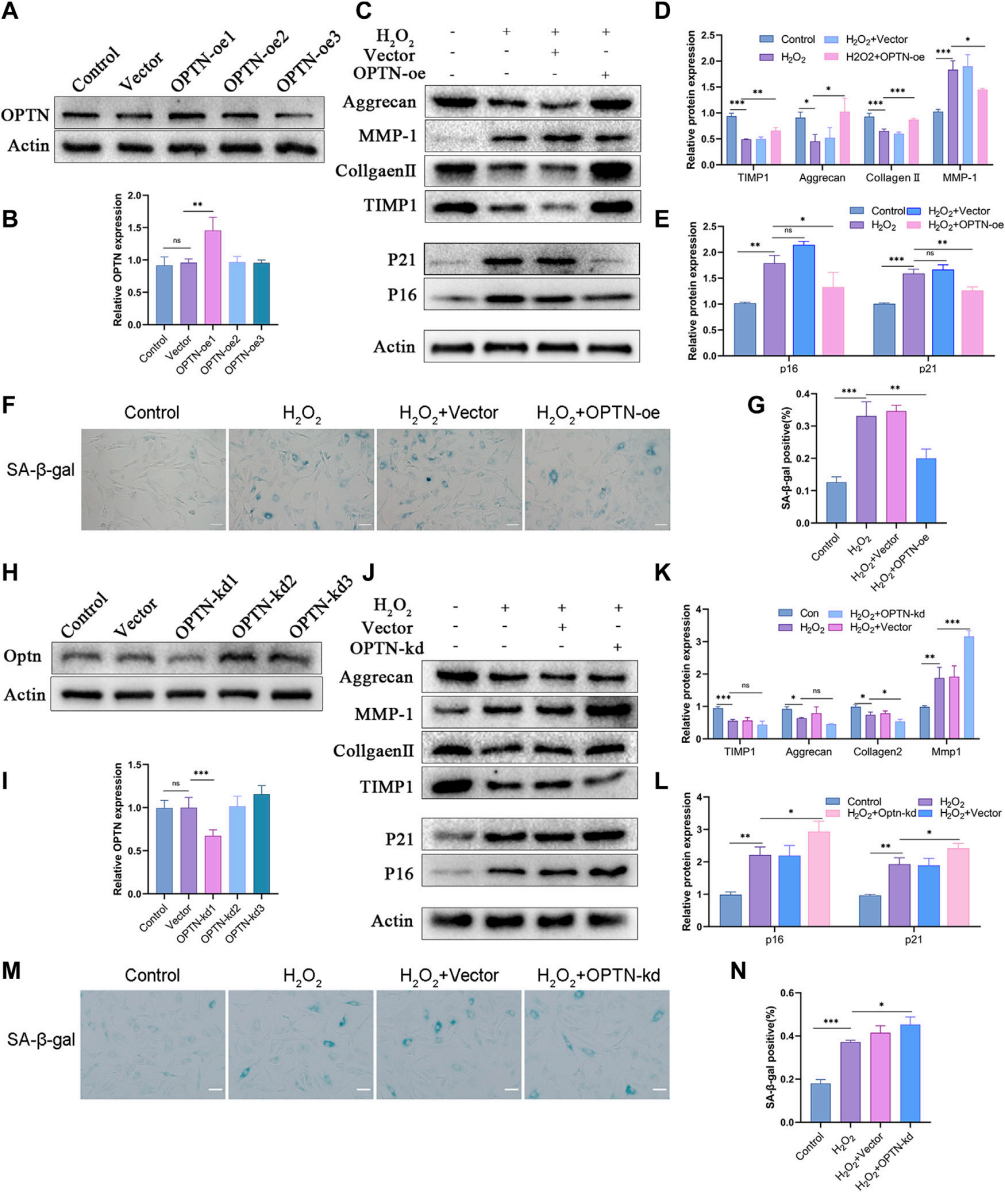
OPTN expression affects senescence and ECM homeostasis of H_2_O_2_-teated rat NPCs. **(A–B)** The expression of OPTN after same overexpression lentivirus transfection with different MOI (OPTN-oe1:15, OPTN-oe2:30, OPTN-oe3:60). **(C–E)** Western blot and quantification of Aggrecan, MMP-1, Collagen Ⅱ, TIMP1, P21, P16 in overexpression lentivirus transfected rat NPCs followed by H_2_O_2_ treatment. **(F–G)** Sa-β-gal staining was used to detect the senescence of overexpression lentivirus transfected NPCs followed by H_2_O_2_ treatment (scale bar: 50 μm). **(H–I)** The expression of OPTN after knockdown lentivirus transfection. **(J–L)** Western blot and quantification of Aggrecan, MMP-1, Collagen Ⅱ, TIMP1, P21, P16 in knockdown lentivirus transfected rat NPCs followed by H_2_O_2_ treatment. (M–N) Sa-β-gal staining was used to detect the senescence of knockdown lentivirus transfected NPCs followed by H_2_O_2_ treatment. All experiments were performed as means ± SD of 3 times in duplicates (scale bar: 50 μm). *p < 0.05, **p < 0.01, ***p < 0.001, ns *p* > 0.05.

Since the change in OPTN expression in human and rat NP tissues was the opposite of that in H_2_O_2_-treated rat NPCs, we knocked down and overexpressed OPTN to investigate the effect of OPTN on H_2_O_2_-treated NPCs. First, we overexpressed and knocked down OPTN in rat NPCs (Figures 2A,B,H,I). We then examined the cellular levels of senescence and matrix homeostasis proteins. The Western blot results indicated that OPTN knockdown increased the expression of P16 and P21 and impaired matrix homeostasis in NPCs (Figures 2C, D, E), whereas OPTN overexpression suppressed the expression of P16 and P21 under H_2_O_2_-induced oxidative stress and reversed the disruption of matrix homeostasis caused by OPTN knockdown (Figures 2J, K, L). SA-β-gal staining was used to examine cellular senescence. Compared with empty vector transfection, OPTN overexpression vector transfection reduced the percentage of SA-β-gal-positive NPCs, and this change was reversed by OPTN knockdown (Figures 2F,G, M, N). These results show that OPTN protects against oxidative stress in NPCs.

### Mitophagy inhibits senescence and matrix degradation in NPCs

**FIGURE 3:**
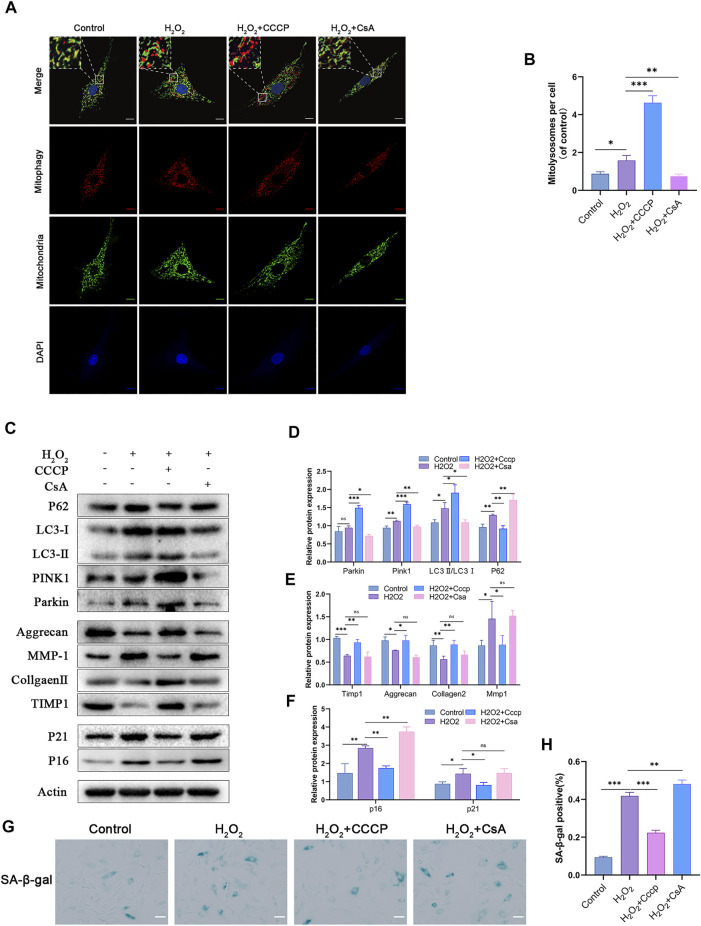
Mitophagy inhibits H_2_O_2_-induced senescence and ECM degradation in rat NPCs. **(A–B)** MITO-QC was used to detect mitolysosomes in rat NPCs and quantification (scale bar: 10 μm). NPCs were treated with or without H_2_O_2_ conditions in the presence of CCCP, CsA. **(C–F)** Western blot and quantification of P62, LC3, PINK1, Parkin, Aggrecan, MMP-1, Collagen Ⅱ, TIMP1, P21, P16 in rat NPCs. **(G–H)** Sa-β-gal staining and quantification (scale bar: 50 μm). All experiments were performed as means ± SD of 3 times in duplicates. (*n* = 10). *p < 0.05, **p < 0.01, ***p < 0.001, ns *p* > 0.05.

Since OPTN acts as a bridging protein in mitophagy, we hypothesized that its role in inhibiting NPCs senescence and maintaining matrix homeostasis might be related to mitophagy. The mitophagy activator CCCP and inhibitor CsA were added to H_2_O_2_-treated NPCs, and changes in mitophagy were observed. The number of mitolysosomes was considerably increased in the CCCP group compared with the H_2_O_2_ group, while CsA had the opposite effect (Figures 3A,B). As shown in Figures 3C,D, the Parkin, PINK1, and LC3II/LC3Ⅰ ratios were notably higher in the CCCP group, while the P62 ratio was significantly lower. CsA had the opposite effects. CCCP activated mitophagy in H_2_O_2_-treated NPCs, while CsA inhibited mitophagy. The levels of the senescence-related proteins P16 and P21 and the percentage of SA-β-gal-positive cells were significantly decreased in the CCCP group compared with the H_2_O_2_ group, and matrix homeostasis was significantly restored in the CCCP group, indicating that CsA inhibited the protective effect of OPTN, increased the expression of senescence-related proteins, and promoted matrix degradation (Figures 3C,E–H). Taken together, these results suggest that H_2_O_2_-induced NPCs senescence and matrix degradation can be reduced by activating mitophagy.

### Mitophagy attenuates NPCs damage by scavenging mtROS

**FIGURE 4:**
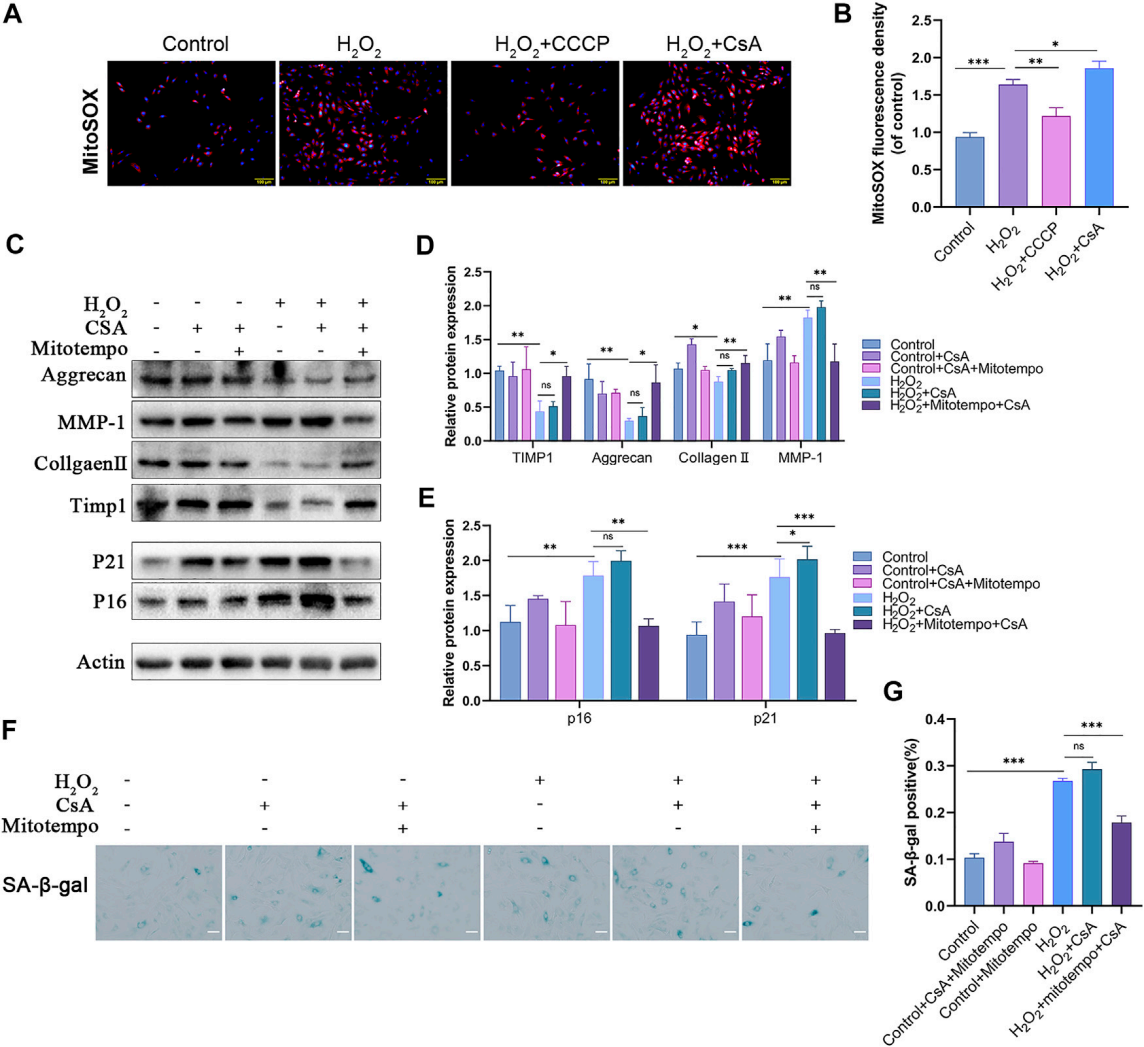
Mitophagy attenuates H_2_O_2_-induced rat NPCs damage by scavenging mtROS. **(A–B)** MitoSOX staining was used to detect mtROS in rat NPCs (scale bar: 50 μm). NPCs were treated with or without H_2_O_2_ conditions in the presence of CCCP, CsA. **C–E**) Western blot and quantification of Aggrecan, MMP-1, Collagen Ⅱ, TIMP1, P21, P16 in rat NPCs. NPCs were treated with or without H_2_O_2_ conditions in the presence of mitoTEMPO, CsA. **(F–G)** Sa-β-gal staining and quantification (scale bar: 50 μm). All experiments were performed as means ± SD of 3 times in duplicates. (*n* = 10). *p < 0.05, **p < 0.01, ***p < 0.001, ns *p* > 0.05.

H_2_O_2_ induces oxidative stress in NPCs (Supplementary Figure 1C), and mitochondria are damaged by this process, resulting in the production of a large amount of mtROS. Mitophagy, as the main process by which injured mitochondria are cleared *in vivo*, plays an important role in scavenging mtROS. We examined the correlation between mitophagy and mtROS levels in NPCs in response to H_2_O_2_ treatment. The results indicated that the fluorescence intensity of the mtROS indicator MitoSOX was significantly reduced in the presence of CCCP and that the inhibitor had the opposite effect (Figures 4A,B). This finding proves that mitophagy deficiency caused by H_2_O_2_ is the main cause of mtROS generation. To further verify the relationship between mtROS and NPCs senescence and matrix degradation, we assessed changes in NPCs using the mtROS scavenger mitoTEMPO. The results confirmed that the mtROS scavenger could effectively inhibit H_2_O_2_-induced cell senescence and matrix degradation. Furthermore, mitoTEMPO mitigated CsA-induced cellular senescence and matrix degradation (Figures 4C–G). These results suggest that mitophagy attenuates the damage to NPCs caused by H_2_O_2_ by scavenging mtROS.

### OPTN attenuates mtROS levels by activating mitophagy and reducing NPCs senescence and matrix damage

**FIGURE 5:**
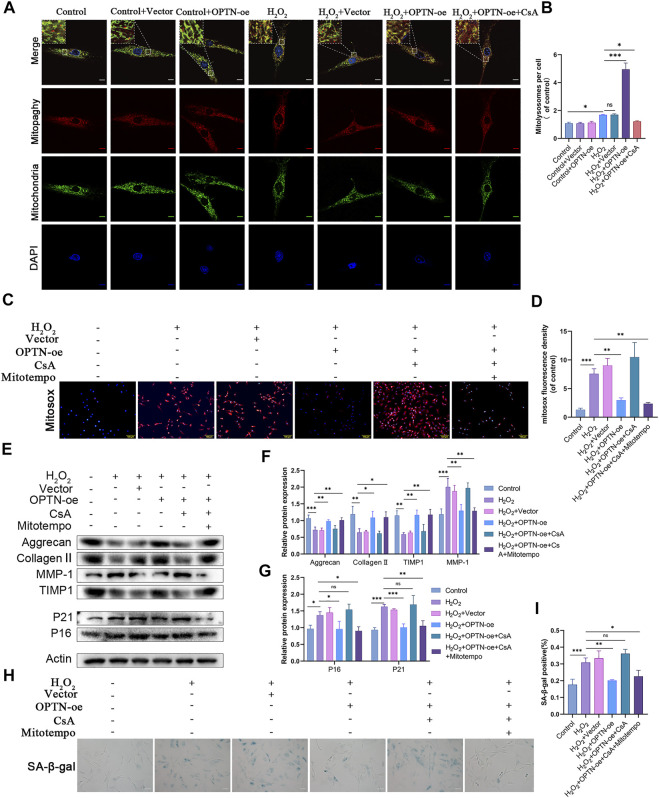
OPTN attenuated mtROS through activation of mitophagy and thus reduced senescence and ECM degradation in NPCs. **(A–B)** MITO-QC was used to detect mitolysosomes in rat NPCs and quantification (scale bar: 10 μm). NPCs were treated with or without H_2_O_2_ conditions in the presence of CCCP, CsA, and OPTN lentivirus transfection. **(C–D)** MitoSOX staining was used to detect mtROS in rat NPCs (scale bar: 100 μm). **(E–G)** Western blot and quantification of Aggrecan, MMP-1, Collagen Ⅱ, TIMP1, P21, P16 in rat NPCs. NPCs were treated with or without H_2_O_2_ conditions in the presence of mitoTEMPO, CsA. (H–I) Sa-β-gal staining and quantification (scale bar: 50 μm). All experiments were performed as means ± SD of 3 times in duplicates. (*n* = 10). *p < 0.05, **p < 0.01, ***p < 0.001, ns *p* > 0.05.

To demonstrate that OPTN mitigates cellular senescence and matrix degeneration by scavenging mtROS through mitophagy, we performed further experiments. As shown in Figures 5A,B, the number of mitolysosomes was significantly increased in the OPTN overexpression group compared with the H_2_O_2_ group, indicating that OPTN, as a mitophagy receptor, can significantly enhance mitophagy. As shown in Figures 5C,D, we observed that the fluorescence intensity of MitoSOX in OPTN overexpression vector-transfected NPCs was significantly reduced compared with that in empty vector-transfected NPCs, while the fluorescence intensity of MitoSOX was increased by the addition of CsA, demonstrating that OPTN inhibited the production of mtROS by enhancing mitophagy. Figures 5E–I show that compared with the empty vector-transfected group, the OPTN overexpression vector-transfected group showed a significant reduction in the percentage of Sa-β-gal-positive cells after H_2_O_2_ treatment; decreased expression of P16, P21, and MMP-1; and enhanced expression of Aggrecan, Collagen II, and TIMP1. These changes were inhibited by the addition of CsA; however, the effect of CsA was reversed by the addition of mitoTEMPO. These results demonstrate the mechanism by which OPTN scavenges MitoSOX by enhancing mitophagy, thereby mitigating cellular senescence and matrix degeneration.

### OPTN upregulation alleviates IVDD in rats

**FIGURE 6:**
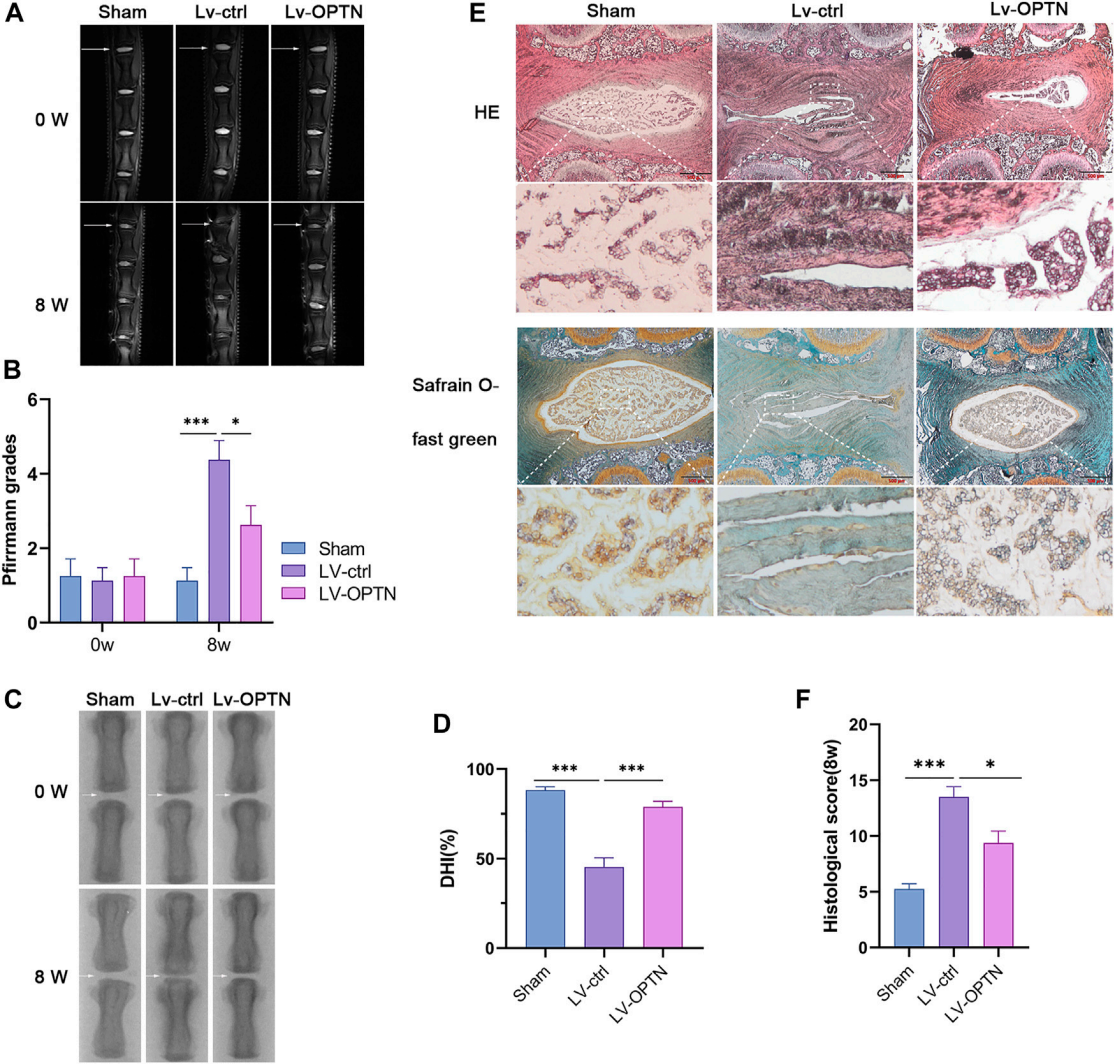
Upregulation of OPTN alleviated IVDD in rats. **(A–B)** MRI and Pfirrmann grade of rat tail discs at 0 and 8 weeks after surgery (white arrows). **(C–D)** The X-ray and DHI% of rat tail discs at 8 weeks after surgery. **(E–F)** Representative HE, Safranin O-fast green staining of NP tissues and histological grades evaluated at 8 weeks in each group. All experiments were performed as means ± SD of 3 times in duplicates. (*n* = 10). *p < 0.05, **p < 0.01, ***p < 0.001, ns *p* > 0.05.

To investigate the effect of OPTN in a rat model of IVDD, we used sagittal T2-weighted magnetic resonance images, and histological scores and measured the intervertebral space height (DHI%) to assess the degree of IVDD in rats at 8 weeks after surgery. OPTN expression in each group was measured by Western blotting. The OPTN expression level was significantly higher in the Lv-OPTN group than in the other two groups (Supplementary Figure S1E). The results indicated that the sagittal T2-weighted MRI signal in the caudal disc was higher and the Pfirrmann grade was lower in the Lv-OPTN group than in the Lv-ctrl group (Figures 6A,B). The DHI% was restored to near normal levels in the Lv-OPTN group compared to the Lv-ctrl group (Figures 6C,D). The H&E staining and safranin O-fast green staining results confirmed the therapeutic effect of OPTN (Figures 6E,F). H&E staining showed that NP tissue in the sham-operated group was large and occupied most of the space in the disc. The surrounding AF was more organized in the sham-operated group than in the Lv-ctrl group. The cells in the NP were stellate and evenly dispersed in a proteoglycan-rich matrix. However, in the Lv-ctrl group, NP tissue was reduced in size and surrounded by a disorganized AF, with cells aggregated in clusters and separated by a streaked proteoglycan matrix, indicating severe degeneration of NPCs. These morphological changes in NP tissue and disintegration and fibrosis of disc structures were significantly alleviated in the Lv-OPTN group compared to the Lv-ctrl group. NP tissue was stained with safranin O in the sham-operated group, indicating the presence of a large amount of proteoglycan matrix. Substantial proteoglycan loss was observed in the Lv-ctrl group compared with the sham-operated group, and the Lv-OPTN group showed less proteoglycan loss. These results confirm our *in vitro* findings that OPTN upregulation increases the ability of NPCs to resist injury and attenuates the severity of IVDD.

## Discussion

The pathogenic mechanisms of IVDD have not yet been fully elucidated. The pathogenesis of IVDD involves multiple factors, and NPCs senescence and an imbalance in matrix homeostasis play essential roles in the progression of this disease. This study demonstrates that H_2_O_2_ contributes to the progression of disc degeneration by impairing mitochondrial function, inhibiting mitophagy and promoting NPCs senescence and the disruption of matrix homeostasis. In addition, OPTN inhibits mtROS, attenuates NPCs senescence, and promotes the restoration of matrix homeostasis by enhancing mitophagy. In conclusion, our findings suggest that enhancing mitophagy by targeting OPTN may be a potential strategy for treating disc degeneration.

We first examined changes in OPTN expression in human and rat NP tissues. Next, we used a model of H_2_O_2_-induced cellular oxidative stress, which is widely used in the intervertebral disc field (Wang Y et al., 2018; Xia et al., 2019; Mei et al., 2021; Shi et al., 2021). We identified differences in the expression of OPTN during the development of IVDD and changes in the expression of OPTN in NPCs subjected to oxidative stress and explored the impact of changes in OPTN expression on NPCs and the potential for OPTN as a therapeutic target for IVDD. We found that the expression of OPTN markedly decreased as IVDD was exacerbated, which is consistent with findings related to the expression of OPTN in degenerative diseases (Cao et al., 2021) and the expression of other mitophagy-related genes in the intervertebral disc (Chen et al., 2020; Hu et al., 2021a). OPTN expression was substantially increased in cells treated with H_2_O_2_ compared with untreated control cells. This result is inconsistent with the findings of Zhang et al. (Zhang et al., 2021), who found that continuous administration of low concentrations of H_2_O_2_ decreases OPTN expression (Zhang et al., 2021); we believe this difference is due to differences in the cell types studied, the concentration of H_2_O_2_ and the duration of treatment. We speculate that short-term oxidative stress causes cells to undergo stress, which promotes mitophagy to counteract the damaging stimulus, resulting in elevated OPTN expression, whereas long-term stress causes oxidative and antioxidant dysregulation, resulting in mitophagy failure. Moreover, OPTN is not the only protein involved in protection against mitochondrial damage, and other proteins, such as NRF2 (Hu et al., 2021b), Sirt3 (Wang J et al., 2018) and Parkin (Zhang et al., 2018), are also involved in mitochondrial damage-related responses. Therefore, different cells may activate different types of proteins to regulate oxidative stress. We confirmed the effect of OPTN on cellular senescence and matrix homeostasis in NPCs and found that OPTN overexpression attenuated H_2_O_2_-induced senescence and matrix degradation in NPCs, while knockdown reversed this effect, suggesting that OPTN enhances the ability of NPCs to respond to antioxidant damage.

There is increasing evidence that mitophagy is involved in IVDD (Wang Y et al., 2018; Chen et al., 2019; Xie et al., 2019; Huang et al., 2020; Lin et al., 2020; Lin et al., 2021b). However, the mechanism is still not fully understood. In our study, we found that Parkin and PINK1 levels and the LC3II/LC3Ⅰ ratio were slightly increased in H_2_O_2_-treated NPCs compared with control NPCs, while the P62 level was significantly increased. We administered the mitophagy inhibitor CsA and activator CCCP and found that CCCP significantly increased Parkin and PINK1 levels and the LC3II/LC3Ⅰ ratio and significantly decreased the P62 level, while CSA showed the opposite effects. The Mito-QC results revealed a change in mitophagy that was consisted with the change revealed by Western blotting. Transient stimulation with H_2_O_2_ increased mitophagy and inhibited the degradation of mitochondrial autophagosomes, resulting in relative inhibition of mitochondrial autophagic flow, while the addition of CCCP significantly increased mitochondrial autophagic flow. Moreover, the levels of the aging-related markers such as P16, P21, Sa-β-gal, and MMP-1, which are related to matrix degradation, showed opposite changes as mitophagy, while the levels of Aggrecan, TIMP1, and Collagen II, which are related to matrix synthesis, showed the same changes as mitophagy. Previous studies have shown that mitophagy clears damaged mitochondria and inhibits mtROS production, which is consistent with our results. Lin et al. showed that LRRK2 knockdown attenuates apoptosis in NPCs by activating mitophagy under oxidative stress (Lin et al., 2021b). Chen et al. reported that Mfn2 overexpression promotes ROS-dependent mitophagy to inhibit IVDD (Chen et al., 2020).

We confirmed that PINK1/Parkin-mediated mitophagy is a potential therapeutic target for IVDD. OPTN is known to play an important role in eliminating damaged mitochondria by enhancing mitophagy (Qiu et al., 2021). Our study showed that OPTN overexpression significantly enhanced mitophagy in H_2_O_2_-treated NPCs, whereas mitophagy was significantly inhibited by the addition of CsA. We further found that senescence was significantly inhibited and matrix synthesis was promoted in OPTN-overexpressing NPCs compared with H_2_O_2_-treated NPCs, whereas the protective effect of OPTN was inhibited by the addition of CsA. Furthermore, the inhibitory effect of CsA was abolished by the mtROS scavenger mitoTEMPO, indicating that OPTN-mediated mitophagy inhibited NPCs senescence and promoted matrix synthesis by scavenging mtROS. This finding suggests that OPTN may be a new therapeutic target for IVDD.

Based on the results of the *in vitro* experiments, we then investigated the role of OPTN *in vivo* and established a rat model of needle puncture-induced disc degeneration. We performed MRI and Pfirrmann grading at 0 and 8 weeks after needle puncture to assess IVDD. As shown in Figure 6, the Lv-OPTN group had a higher T2-weighted signal intensity and lower Pfirrmann grade than the Lv-ctrl group. The protective effects of OPTN were further confirmed by H&E staining and safranin O fast green staining of rat disc tissues at 8 weeks. H&E staining showed that in the disc of rats in the sham group, NP tissue was complete. NPCs were evenly distributed in the extracellular matrix, and the AF tissue around the NP was clearly structured and had good continuity with the NP tissue. Compared with that in the control group, NP tissue in the Lv-ctrl group was significantly atrophied, with poor continuity with the AF and structural breakage of AF tissue. In contrast, OPTN treatment significantly reduced the disruption of disc structure and fibrosis in NP tissue. Safranin O green staining, which represents the proteoglycan matrix, was significantly reduced in the Lv-ctrl group compared with the sham group. However, these tissues were partially preserved in the Lv-OPTN group compared with the Lv-ctrl group. These results demonstrate that OPTN protects against IVDD.

However, our study is still limited in several ways. First, *in vivo* NPCs are exposed to a significantly more complex environment than the *in vitro* simulated oxidative stress environment, and the *in vitro* model cannot fully simulate the complex *in vivo* environment. Second, the exact mechanism by which oxidative stress alters OPTN expression was not confirmed. Our *in vivo* experiments were performed only in rats, and experimental studies in large animals are still lacking. In addition, whether OPTN affects IVDD through mechanisms other than mitophagy needs further investigation.

## Conclusion

In conclusion, our study shows that OPTN is differentially expressed in human and rat NP tissues and in H_2_O_2_-treated rat NPCs *in vitro*. Furthermore, OPTN protects NPCs against oxidative stress-induced cellular senescence and matrix degradation. OPTN protects NPCs against oxidative stress by enhancing mitophagy and scavenging mtROS, thereby reducing cellular senescence and restoring matrix homeostasis. To the best of our knowledge, this is the first report to explore the role of OPTN in the mechanisms of IVDD. These findings may provide potential targets for the treatment of IVDD.

## Data Availability

The original contributions presented in the study are included in the article/Supplementary Material, further inquiries can be directed to the corresponding author.
