# Supplementary material for: Optineurin-mediated mitophagy as a potential therapeutic target for intervertebral disc degeneration
Source: Front Pharmacol. 2022 Aug 29;13:893307. doi: 10.3389/fphar.2022.893307 (PMC9465714; doi:10.3389/fphar.2022.893307)
Supplement: Supplementary file 1 [file DataSheet1.docx]

Supplementary Material

## Supplementary Figures


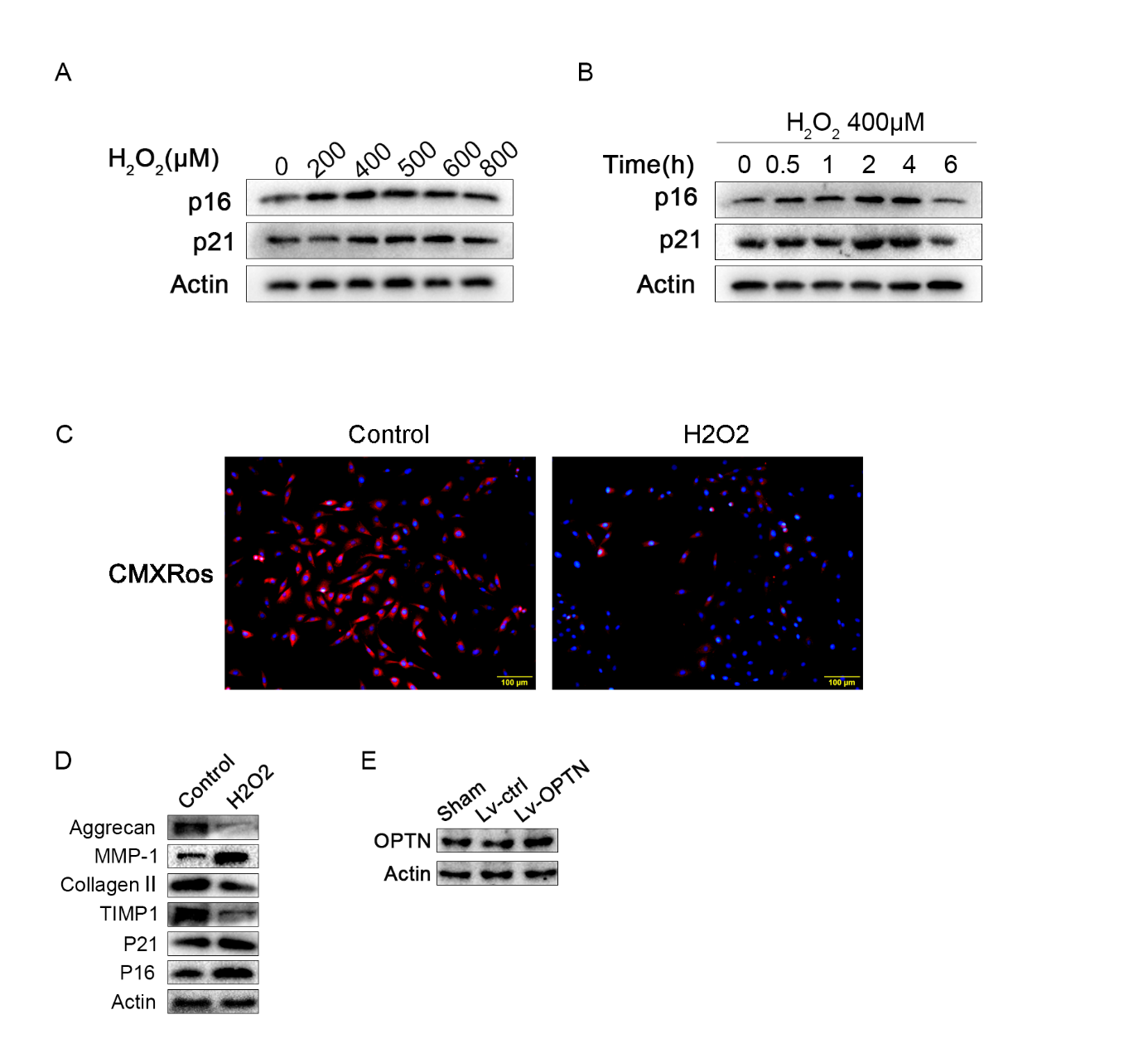


**Supplementary Figure 1.** (A) The protein expression of P16, P21 in rat NPCs treated with different concentrations of H_2_O_2_ for 2 h. (B) The protein expression of P16, P21 in rat NPCs treated with 400μΜ H_2_O_2_ for different times. (C) MitoTracker red CMXRos staining of rat NPCs treated with 400μΜ H_2_O_2_ for 2h (scale bar: 100μm). (D) The protein expression of P16, P21, MMP-1, CollagenⅡ，TIMP1, Aggrecan in rat NPCs treated with 400μΜ H_2_O_2_ for 2h. (E) The OPTN expression of each group in vivo. All experiments were performed as means ± SD of 3 times in duplicates.
